# A Comparative Reverse Docking Strategy to Identify Potential Antineoplastic Targets of Tea Functional Components and Binding Mode

**DOI:** 10.3390/ijms12085200

**Published:** 2011-08-15

**Authors:** Rong Zheng, Tuan-sheng Chen, Tun Lu

**Affiliations:** 1 Institute of Biological Science and Engineering, Fuzhou University, Fuzhou, Fujian 350108, China; E-Mail: cogcoz@gmail.com; 2 Institute of Life Sciences, Fujian Agriculture and Forestry University, Fuzhou, Fujian 350002, China; E-Mail: cts129@163.com; 3 Fujian Supercomputer Center, Fuzhou, Fujian 350108, China

**Keywords:** tea polyphenols, reverse docking, target protein, binding mode, virtual screening

## Abstract

The main functional components of green tea, such as epigallocatechin gallate (EGCG), epigallocatechin (EGC), epicatechin gallate (ECG) and epicatechin (EC), are found to have a broad antineoplastic activity. The discovery of their targets plays an important role in revealing the antineoplastic mechanism. Therefore, to identify potential target proteins for tea polyphenols, we have taken a comparative virtual screening approach using two reverse docking systems, one based on Autodock software and the other on Tarfisdock. Two separate in silico workflows were implemented to derive a set of target proteins related to human diseases and ranked by the binding energy score. Several conventional clinically important proteins with anti-tumor effects are screened out from the PDTD protein database as the potential receptors by both procedures. To further analyze the validity of docking results, we study the binding mode of EGCG and the potential target protein Leukotriene A4 hydrolase in detail. We indicate that interactions mediated by electrostatic and hydrogen bond play a key role in ligand binding. EGCG binds to the enzyme with certain orientation and conformation that is suitable for nucleophilic attacks by several electrical residues inside the enzyme’s activity cavity. This study provides useful information for studying the antitumor mechanism of tea’s functional components. The comparative reverse docking strategy presented generates a tractable set of antineoplastic proteins for future experimental validation as drug targets against tumors.

## 1. Introduction

Green tea is a popular beverage worldwide, and it has been found to have a relatively strong antineoplastic effect. The main active components of green tea are tea polyphenols composed of multiple catechins, such as EGC, ECG, EC and mainly EGCG. Numerous studies have shown that ECGC has a strong, complicated anti-tumor effect, including inhibiting tumor growth and inducing apoptosis, as well as inhibiting multi-targets growth, AKT/protein kinase B signaling pathway and so on. But the detailed mechanism is still unclear. Recent researches have indicated that cell signal transduction is bound up with tumor transformation and development [1]. In signal transduction pathways, the interactions between tea polyphenols, a signal transduction molecule, and their target receptors play a key role in cancer prevention. Moreover, designing targeted drugs for key receptor molecules in signal transduction pathways is a promising method for tumor therapy.

However, although researchers have been looking for EGCG receptors over the years, possible receptors remain a big controversy nowadays. Recent studies have reported that EGCG can bind to the 67 kDa laminin receptor (67LR) related to cancerometastasis [2,3], but Adachi *et al*. indicate that EGCG may not directly bind to the cell surface receptor [4]. In contrast to EGCG, studies on ECG, EGC and EC have rarely been reported, especially regarding their biological activity mechanism. All of these studies still need further investigation by large-scale randomized trials.

Therefore, how to search for the molecular targets of tea polyphenols has effectively become an important and challenging task. Using proteomic profile and pharmacokinetic approach to identify the drug targets among thousands of candidate macromolecules has been proved to be laborious and time-consuming [5–8]. Therefore, some researchers resort to computing methods such as virtual screening to facilitate the experiment. However, the traditional virtual screening based on receptors usually contains two parts: the docking procedure and a drug molecular database. With the increased number of known protein structures, a new docking method called reverse docking was developed, in which docking is carried out by searching a protein database instead of a drug molecular database. For example, Chen, *et al*. successfully developed INVDOCK [9], a reverse docking system to study drug toxicity. Li, *et al*. developed a reverse docking web server, Tarfisdock [10], and used it to identify drug targets. These pioneering researches indicate that reverse docking procedures can be used to identify the target proteins of tea polyphenols. The above-mentioned reverse docking systems are both based on the widely used docking program, DOCK, and use ligand-protein interaction energy to assess the docking results [11]. Yet another popular docking program, Autodock [12], which employed a more precise score function and different algorithms, has not been tried for this purpose. A number of studies have proved that the ligand-protein binding mode predicted by Autodock agrees well with experimental results [13–16]. So Autodock could also be used for reverse docking purposes and we develop a reverse docking system based on it in this research. As to the protein database, all the records in PDB database are original experimental data, they cannot be directly used for docking calculations, therefore secondary databases must be developed for docking programs, such as the Potential Drug Target Database PDTD [17], a comprehensive network database that contains more than 1100 3D protein structures and covers 15 therapeutic areas including tumor therapy.

In this research, we take a comparative virtual screening approach which uses both the reverse docking system based on Autodock and the Tarfisdock system, to identify potential target proteins for tea polyphenols. Two separate in silico workflows are implemented to derive a set of protein targets ranked by the energy score from PDTD database, and all of the derived targets are related to human diseases, including tumors. To analyze the validity of docking results, we further explore the binding mode between tea polyphenols and their potential targets. This study may provide useful information for studying the antitumor mechanism of tea functional components. Our work also helps to design new targeted drugs with strong antineoplastic effects by providing the information about the binding mechanism of tea polyphenols to key putative receptor proteins.

## 2. Results and Discussion

**Figure 1.**
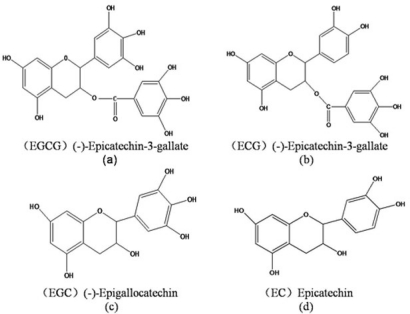
Structure of tea functional components EGCG, ECG, EGC and EC.

Tea functional components studied in this work include epigallocatechin gallate (EGCG), epicatechin gallate (ECG), epigallocatechin (EGC) and epicatechin (EC). Their chemical structures are displayed in Figure 1.

### 2.1. Potential Protein Targets for EGCG

Potential protein receptors for EGCG identified by our virtual screening procedures compared with experiment data are listed in Table 1. Computed binding free energies and experimental references for several EGCG-protein complexes are also included. The result shows that this strategy does screen out several EGCG receptors, including various types of cancer or other diseases related receptors. In addition, five out of nine protein receptors verified by previous experimental report are discovered by virtual screening.

Fourteen potential receptors whose binding energies rank in the top 3%–2% by Autodock and Tarfisdock, respectively are listed in Table 1. Among them, eight receptors are screened out by Autodock alone and three by Tarfisdock alone. Three receptors, HIV protease, Leukotriene A4 and Farnesyl protein transferase are screened out by both software. To our delight, HIV protease is a valuable target of EGCG which has been implicated experimentally, and Leukotriene A4 and Farnesyl protein transferase are both likely to be important targets for EGCG. Four potential protein receptors, glutathione reductase, catalase, cholesterol oxidase and eEF1-α identified by Autodock have supporting experimental evidence. Anti-oxidation experiments in vivo have shown that EGCG plays a protective and prothetic role of intracellular GSH [19,20] and catalase [22]. It also interacts with cholesterol enzymes to inhibit the absorption of cholesterol [21]. A recent report indicates that silencing of eEF1-α in tumor cells results in the abrogation of EGCG-induced tumor growth inhibition *in vivo* [3]. It is of interest that many predicted EGCG-binding proteins are associated with cancers or tumors. For example, Histone deacetylase is perceived as a new important antitumor target in recent years; and some experimental results indicate that the inhibition of prostagladin D synthase may improve the symptoms of prostate cancer cachexia [26] and the expression of TGFβ-R-I can act as an indicator of renal carcinoma [27]. However, more experiments are needed to verify EGCG binding targets found in our work.

Some proteins which might react with EGCG according to experimental data, such as 67 kD laminin receptor, ZAP-70 and Fyn kinase, are not found in the top 3% rank by our screening system, but they also have weak binding-active with EGCG (see Table 1). The main reason for the absence of these potentailreceptors may be that both Autodock and Tarfisdock do not take the current flexibility of protein conformation into account.

### 2.2. Potential Protein Receptors for ECG, EGC and EC

The structures of tea functional components other than EGCG, such as ECG, EGC and EC are similar to EGCG. They may also possess certain biological activity. Therefore, virtual screening of their potential protein receptors is also performed. Co-receptors of these four tea polyphenols are listed in Table 2, which rank in top 2% of the targets identified by Autodock sorted by their binding energies. We note that some familiar clinical protein targets can bind to all of the 4 types of catechins according to this list, such as Cytochrome reductase P450 and Oxidosqualene cyclase. It indicates that ECG, EGC, and EC may also have the same anti-tumor effect as EGCG and could reduce cholesterol to some degree. It suggests that catechins may act on cytokine and key drug metabolism enzymes that involve in steroid hormone biosynthesis. They may have a potential effect on hypercholesterolemia, cardiac disease, and various inflammatory diseases. These results also need further experimental evidence Besides this, voltage-gated potassium channels, phospholipase A2, FK506 tacrolimus binding protein, and other several receptors can also bind to catechins to some extent. In addition, the structure between ECG and EGCG differs by only one hydroxyl group on the benzene ring, therefore they have relatively more co-receptors.

### 2.3. Comparing Screening Results of Autodock and Tarfisdock

Autodock and DOCK are both famous free software widely used in drug design field. Based on DOCK, Tarfisdock now works as a web server which is more convenient for screening drug targets than Autodock. As mentioned above, the two molecular docking procedure Autodock and DOCK have their respective characteristics. Autodock uses Lamarckian genetic algorithm [12] and a more comprehensive scoring function which takes into account of hydrogen bonding and solvation free energy [28], while DOCK uses Fragment growth method (Systemic searching method) and a scoring function composed of non-bond interaction energy terms [10,11] which only takes van der Waals interaction and electrostatic interaction into consideration. For these reasons, Tarfisdock finishes the whole protein database search within 19–28 h depending on the flexibility of tea polyphenols, while Autodock should need 27–33 days in this work. To make our result more reliable, we compare virtual screening results of Autodock and DOCK to get common receptors for tea polyphenols found by both methods.

Potential receptors for tea polyphenols identified by both procedures are listed in Table 3. According to the results listed in the table, the best EGCG-binding receptors are Leukotriene A4 hydrolase, farnesyl protein transferase (FPT) and HIV protease *etc*. EGC most likely combines with FKBP tacrolimus protein, glutamate carboxypeptidase II, *etc*. Squalene oxide cyclase and Leukotriene A4 hydrolase are the most potential protein targets for ECG. Squalene oxide cyclase is the only receptor for EC recognized by both Autodock and Tarfisdock.

The screening results of the 4 types of catechins by two different software are different. EGC has the highest match rate, the number of common receptors divide by total number of receptors found, with match rate of 27.30%, EGCG followed by 18.20%, EC only by 4.50%. The difference is mainly due to the different receptor pockets-determining methods, conformational search algorithms and energy scoring functions used by these two software.

The binding free energies between protein and ligand are calculated by an energy scoring function in Autodock as shown by Equation 1.

(1) ΔG = ΔGvdw∑i,j(Aijrij12−Bijrij6) + ΔGhbond∑i,jE(t)(Cijrij12−Dijrij10) + ΔGele∑i,jqijɛ(r)rij+ ΔGtorNtor + ΔGsol∑i,j(SiVj+SjVi)e(−rij22δ2)

In the above equation, the total binding energy is composed of van der Waals energy, hydrogen bond energy, electrostatic energy, ligand rotary energy and solvation free energy. In contrast, Tarfisdock only focuses on the interaction energies whose energy function is shown by Equation 2.

(2) $$E_{\text{inter}}=\sum_{i=1}^{\text{lig}}\sum_{j=1}^{\mathrm{rec}}(\frac{A_{\mathrm{ij}}}{r_{\mathrm{ij}}^{a}}-\frac{B_{\mathrm{ij}}}{r_{\mathrm{ij}}^{b}}+332.0\;\frac{q_{i}q_{j}}{{\mathrm{Dr}}_{\mathrm{ij}}})$$

In Equation (2), the total energy is composed of van der Waals energy and electrostatic energy. In both Equation (1) and (2), each term is a double sum over ligand atoms *i* and receptor atoms *j*; *r_ij_* is the distance between atom *i* and atom *j*; *A_ij_*, *B_ij_*, *C_ij_* and *D_ij_* are van der Waals repulsion and attraction parameters, respectively; a and b are the van der Waals repulsion and attraction exponents, respectively; *q_i_* and *q_j_* are point charges on atoms *i* and *j*; D and *ɛ*(*r*) are dielectric constant; and 332.0 is the factor that converts the electrostatic energy into kcal/mol.

Comparing these two docking procedures’ results, we find that EGCG and ECG share a common best receptor: Leukotriene A4 hydrolase, for its lower binding energy get by both procedures. Therefore, we tried to explore the binding mode between these two kinds of catechins and leukotriene A4 hydrolase.

### 2.4. Docking Results for the Original Ligand BES in Leukotriene A4 hydrolase Crystal Structure

Myocardial infarction is recognized as the world's leading cause of death. Hakonarson, H. *et al*. indicated that mutants correlating with two genes in leukotriene pathway are associated with the risk of myocardial infarction [29]. 13% patients carried Leukotriene A4 (LTA4) hydrolase mutant gene. This hydrolase is composed of a group of bioactive enzymes, whose genetic polymorphism may increase the risk of myocardial infarction. Moreover, it plays an important role in the regulation of low-sensitivity reactions and inflammation. Besides this, its overexpression in esophageal adenocarcinoma cells is also one of the early symptoms of esophageal adenocarcinoma.

**Figure 2.**
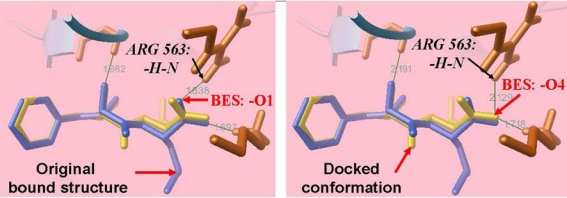
The docked ligand BES compared with its original bound structure in crystallographic complex. It showed that the docked structure is very close to the experimental structure: Blue: original bound structure in crystal complex (left figure); Yellow: the docked conformation (right figure).

LTA4 hydrolase is a kind of zincic metalloprotease with intrinsic aminopeptidase activity, containing 610 amino acid residues, 43 α-helix, 7 β hairpin and 22 β sheet. Although numerous applications have demonstrated the success of Autodock in docking simulations for reproducing experimental binding structures, it remains a question whether it works for the particular biological system of interest. To examine the reliability of the docking method, the original ligand BES from a ligand-LTA4 hydrolase complex structure is removed from the PDB file and docks back to the ligand binding site using the same docking strategy as EGCG. The superposition of the structures of the docking simulation results and that of the crystallographic structure complex is shown in Figure 2. As shown in Figure 2, Arg563 forms a hydrogen bond (O…H—N) with the oxygen atom O1 of the original bound ligand and O4 of the docking conformation respectively. Nonetheless, LTA4 hydrolase forms 3 hydrogen bonds with the docked conformation similarly to the original bound structure, with the error of bond length within 0.29 Å. The root-mean-square deviations of all heavy atoms of BES between the docked poses and the crystallographic coordinates range from 0.3 to 1.5 Å. Apart from that, the docked pose is overall very close to the experimental structure. These results show that the docking method is able to reproduce the experimental binding structure of ligand-LTA4 hydrolase complex.

### 2.5. Binding Mode between Leukotriene A4 Hydrolase and EGCG

**Figure 3.**
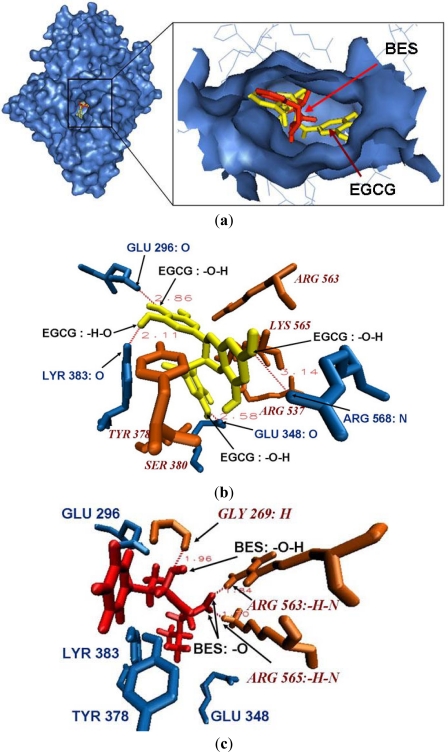
Micro environment of the bind site of EGCG/BES and Leukotriene A4 hydrolase. (**a**) EGCG (yellow) binds to Leukotriene A4 hydrolase in the active cavity (catalytic center and binding domain) where the original ligand BES (red) located; (**b**) Residues GLU348, SER380, TYR383, TYR378, ARG563, ARG565, ARG568, GLU296 and ARG537 make up a electrostatic cavity; hydroxyl groups of EGCG form hydrogen bonds with the surrounding amino acids GLU348, TYR383, GLU296 and ARG568 (blue); (**c**) The original ligand formed 3 strong hydrogen bonds with residues GLY269, ARG563, and ARG565 (orange).

The docking conformations of BES/EGCG and Leukotriene A4 hydrolase complexes predicted by Autodock are given in Figure 3, with the binding free energy −6.8 and −5.22 kcal/mol respectively. As shown in Figure 3(a), EGCG binds to Leukotriene A4 hydrolase in the active cavity where the original substrate BES located. In Figure 3(b) and (c), residues such as TYR383 and GLU296, colored in marine or orange, represent the catalytic center of the leukotriene A4 hydrolase. From Figure 3(b), we can see the binding mode of EGCG and Leukotriene A4 hydrolase. Three benzene ring planes of EGCG are located in the active cavity composed of electrical amino acids such as GLU348, TYR378, SER380, TYR383, ARG568, GLU296, LYS565, ARG563, ARG537, *etc*. On the other hand, polar residues GLU348, TYR383, GLU296 and ARG568, colored in marine, form 3 hydrogen bonds O…O—H and 1 hydrogen bond N…O—H with four hydroxyl groups of EGCG, with bond distance of 2.58 **Å no bold**, 2.11 **Å**, 2.86 **Å** and 3.14 **Å**, respectively. The interaction between Leukotriene A4 hydrolase and its original ligand is shown in Figure 3(c), where residues GLY269, ARG563, ARG565 colored in orange form 1 strong hydrogen bond H…O—H and 2 strong hydrogen bonds N—H…O with ligand BES. The bond distances are 1.96 **Å**, 1.84 **Å**, and 1.70 **Å**, respectively.

Our analysis shows that the interactions mediated by electrostatic and hydrogen bonds play a key role in the binding of EGCG and Leukotriene A4 hydrolase. It suggests that EGCG may bind to leukotriene A4 hydrolase catalytic site through electrostatic and hydrogen bonds interactions, thus prevents the exposure of the catalytic site to the substrate BES and acts as a competitive inhibitor of the enzyme.

## 3. Experimental Section

### 3.1. The Three-Dimensional Structures of Tea Polyphenols and Drug Targets

Crystal structure of the four kinds of tea polyphenols: (EGCG.pdb, EGC.pdb, ECG.pdb, EC.pdb) come from MMsINC database (http://mms.dsfarm.unipd.it/MMsINC/search/ligandsearch.php). Crystal structure of receptors: 1044 kinds of potential drug target molecules are selected from PDTD database (http://www.dddc.ac.cn/pdtd/). Polar hydrogen atoms and Kollman charges are added to all mol2 files of the receptors. All of the potential drug targets in PDTD database are related to cancer, diabetes and other diseases, including 79.8% of the enzymes, 4.6% of the receptor, 3.5% of the monoclonal antibody, 3.3% of the regulatory factors and hormones. Three experimental implicated targets of EGCG that are absent in PDTD are obtained from PDB database.

### 3.2. Reverse Docking Procedure Using Autodock and Tarfisdock

Reverse docking based on Autodock4: we use a reverse docking system by providing a series of cshell scripts independently to invoke Autodock core code. These scripts will combine the docking procedure with a protein database and dock a mall molecule to all proteins in the database one by one automatically. The corresponding receptors of these four mall molecules (EGCG, EGC, ECG, EC) are screened by this system using PDTD as its protein database. Gasteiger charge and all the hydrogen atoms (polar and non-polar) are added to receptors initially. For docking, the grid box size of the receptor molecules is 81 * 81 * 81 Å^3^, the grid spacing is 0.375 Å, the box located at the center of the receptor. The procedure uses optimizing Lamarckian genetic algorithm for global searching and uses Solis & Wets simulated annealing for local searching. Global optimization generates 500 randomly initial conformations for each ligand, the maximum energy evaluation number is 2.5 × 10^7^ and the maximum offspring number is 2.7 × 10^4^. Two independent GA calculations are carried out. For the Solis & Wets simulated annealing setting, the maximum number of iterations is 50, each iteration is set to carry four receive and four refused steps, and the default setting is used for other parameters. The lowest docking energy value is used to assess screening results.

Reverse docking based on DOCK4: Tarfisdock web service (http://www.dddc.ac.cn/tarfisdock/) is used. Virtual screening for potential receptors of tea polyphenols (EGCG, EGC, ECG, EC) is carried out by invoking DOCK4.0 to search PDTD database from this web interface.

## 4. Conclusions

In this study, a comparative reverse docking approach using Autodock and Tarfisdock is taken to identify the potential receptors for green tea bioactive substances such as EGCG, EGC, ECG and EC. The results show that: (1) a number of receptors of EGCG identified by our method such as Glutathione reductase, Catalase and eEF1-α *etc*. have been verified by experiments. While some other potential receptors such as Squalene oxide cyclase, FPTase, Leukotriene A4 hydrolase and FKBP, are either conventional clinical targets with anti-tumor effects or target enzymes of drug design; (2) Several receptors identified by two different docking procedures are proposed as best putative targets for four types of catechin; (3) We further explore the binding mode between the most potential receptor Leukotriene A4 hydrolase and EGCG. We propose that EGCG binds to the Leukotriene A4 hydrolase active site with certain orientation and conformation so that it may acts as a competitive inhibitor of that enzyme. Structure analysis shows that electrostatic interaction and hydrogen bonding play an important role in their binding process. This study provides important information for studying EGCG and its derivatives’ antitumor mechanism and new targeted drugs design.

## Figures and Tables

**Table 1.** EGCG possible targets found by screening procedures compared with experiment.

**PDB_ID**	**Target Name**	**Predicted by Procedures**	**Implicated by Experiment**	**Energy Score (kcal/mol)**	**Reference or Related Disease**
3BCH	67 kD laminin receptor	Autodock	YES	−3.75	[2]
7HVP	HIV protease	Autodock/Tarfisdock	YES	−5.01/−44.87	[18]
1GRE	glutathione reductase	Autodock	YES	−6.80	[19,20]
1IJH	cholesterol oxidase	Autodock	YES	−6.77	[21]
8CAT	catalase	Autodock	YES	−6.23	[22]
1JNY	eEF1-α	Autodock	YES	−5.70	[3]
1BOO	DNA methyltransferase	Autodock	YES	−4.66	[23]
2OZO	ZAP-70	Autodock	YES	−3.81	[24]
2DQ7	Fyn kinase	Autodock	YES	−4.91	[25]
1HS6	Leukotriene A4	Autodock/Tarfisdock	NO	−5.22/−48.2	Esophagus cancer
1FT2	Farnesyl protein transferase	Autodock/Tarfisdock	NO	−4.1/−44.94	Cancer/Tumour
1UTR	Mammalian PCB-binding protein	Autodock	NO	−7.19	Lung Cancer
1JVM	Voltage-Gated Potassium Channel	Autodock	NO	−6.58	Cardiomyopathie
1OG5	CYP450	Autodock	NO	−6.49	Tumour
1VKG	Histone deacetylase	Autodock	NO	−5.63	Tumour
1OOQ	Dihyrofolate reductase	Tarfisdock	NO	−46.42	Tumour
1IYH	Hematopoietic Prostagladin Synthase	Tarfisdock	NO	−44.50	Cancer
1PY5	TGF-beta receptor type I	Tarfisdock	NO	−44.33	Renal carcinoma

**Table 2.** Potential co-receptors of 4 types of Tea-polyphenols screened by Autodock.

**Target Name**	**4 Types of Tea-Polyphenols**			
	**EGCG**	**EGC**	**ECG**	**EC**
CYP450	√	√	√	√
Oxidosqualene cyclase	√	√	√	√
Voltage-Gated Potassium Channel	√	√	—	√
Phospholipase A2	√	√	√	√
FK506 binding protein	√	—	√	√
Dihyrofolate reductase	—	√	√	√
Leukotriene A4 hydrolase	√	—	√	—
PARP(Poly ADP-Ribose Polymerase)	√	√	√	—
Protoporphyrinogen oxidase	√	√	√	—
Ornithine Aminotransferase	—	—	—	√
1,2-Cyclooxygenase	—	—	—	√
histone deacetylases (HDACs)	—	—	√	√
Amino acid oxidase	—	—	√	√
Glutamic acid receptor-2	—	√	—	—
Mammalian PCB-binding protein	√	—	√	—
Fatty acid-binding protein (FABP)	√	—	√	—
Catalase	√	√	—	—

**Table 3.** The most possible targets screened by Autodock and Tarfisdock (top 3%).

**Ligand Name**	**PDB_ID**	**Score Tarfisdock (kcal/mol)**	**Score Autodock (kcal/mol)**	**Receptor Name**
EGCG	1HS6	−48.21	−5.22	Leukotriene A4 hydrolase
	1FT2	−44.94	−4.10	Farnesyl protein transferase
	7HVP	−44.87	−5.01	HIV protease
	1Y79	−44.11	−3.96	Dipeptidyl peptidase
EGC	1YTV	−36.44	−5.66	Vasopressin V1a receptor
	1Q5M	−35.83	−3.32	Alpha hydroxysteroid dehydrogenase
	1NR5	−35.24	−4.01	3-dehydroquinate Synthase
	1Y79	−35.19	−3.77	Peptidyl dipeptidase
	2C6C	−48.27	−3.31	Glutamate carboxypeptidase II
	1TCO	−43.82	−6.07	FK506 binding protein
ECG	1W6K	−38.06	−6.07	Oxidosqualene cyclase
	1HS6	−46.21	−5.74	Leukotriene A4 hydrolase
EC	1W6K	−38.06	−6.07	Oxidosqualene cyclase
